# Characteristics of fatal and hospital admissions for burns in Fiji: A population-based study (TRIP Project-2)

**DOI:** 10.1016/j.burns.2011.11.005

**Published:** 2012-08

**Authors:** Mable Taoi, Iris Wainiqolo, Berlin Kafoa, Bridget Kool, Asilika Naisaki, Eddie McCaig, Shanthi Ameratunga

**Affiliations:** a Research Unit, College of Medicine, Nursing and Health Science, Fiji National University, Suva, Fiji; b Office of the Dean, College of Medicine, Nursing and Health Science, Fiji National University, Suva, Fiji; c Section of Epidemiology and Biostatistics, School of Population Health, The University of Auckland; Auckland, New Zealand; d Department of Public Health & Primary Care, Fiji National University, Fiji; e Surgery (Orthopaedics), College of Medicine, Nursing and Health Science, Fiji National University, Suva, Fiji

**Keywords:** Burns, Epidemiology, Developing countries

## Abstract

**Background:**

Over 95% of burn deaths are estimated to occur in low-and-middle-income countries. However, the epidemiology of burn-related injuries in Pacific Island Countries is unclear. This study investigated the incidence and demographic characteristics associated with fatal and hospitalised burns in Fiji.

**Methods:**

This cross-sectional study utilised the Fiji Injury Surveillance in Hospital database to estimate the population-based incidence and contextual characteristics associated with burns resulting in death or hospital admission (≥12 h) during a 12-month period commencing 1st October 2005.

**Results:**

116 people were admitted to hospital or died as a result of burns during the study period accounting for an overall annual incidence of 17.8/100,000 population, and mortality rate of 3.4/100,000. Most (92.2%) burns occurred at home, and 85.3% were recorded as unintentional. Burns were disproportionately higher among Fijian children compared with Fijian–Indian children with the converse occurring in adulthood. In adults, Indian women were at particularly high risk of death from self-inflicted burns as a consequence of ‘conflict situations’.

**Conclusion:**

Burns are a significant public health burden in Fiji requiring prevention and management strategies informed by important differences in the context of these injuries among the major ethic groups of the country.

## 1 Introduction

Globally, burns represent a major health problem contributing to high mortality, morbidity and economic loss, but the context and risks involved can vary substantially in different population groups. In 2008, fire-related burns were responsible for approximately 300,000 deaths globally, and ranked among the 15 leading causes of deaths and burden of disease in children and young adults aged 5–29 years. Over 95% of these deaths occurred in low-and-middle-income-countries (LMIC) [1]. Not surprisingly, the impact of burns can induce further poverty in already disadvantaged regions [2].

The common causes of burns also vary substantially by region. Fire-related burn mortality rates are reportedly highest in South East Asia (11.6 per 100,000), with considerably lower rates in the LMICs in the Western Pacific region (1.2 per 100,000) [1]. Scalding is the second leading cause of burns in LMICs, a mechanism particularly common in childhood [3].

Increasing knowledge of the burden of these injuries worldwide is providing much-needed impetus for a global agenda on burns prevention. However, country level data are necessary to galvanise action at a local level. In this regard, published research on the epidemiology of burns in the small island nations of the Pacific is scant. Three studies from Papua New Guinea, published more than 15 years ago, focused on grass skirt burns [4], self-inflicted burns among women using kerosene [5], and a 7 year retrospective review of admissions to Port Moresby General Hospital [6]. The latter identified the commonest causes of burns to be hot water scalds (43%), falling into fires, and self-immolation (13%). This study also suggested the risk profiles varied considerably by demographic groups, with children accounting for a third of burns. The potential heterogeneity in the epidemiology of burns within the country is of particular salience in Fiji, where the majority of people are indigenous Fijian (56.8%) but 37.5% are of Indian (South Asian) ethnicity [7]. While the migration experiences of the Indian community are varied, many are descended from indentured migrants recruited to work in the sugar industry from 1879 to 1920.

As part of a larger project funded by the Wellcome Trust (UK) and the Health Research Council of New Zealand (TRIP Project), we established an active prospective injury surveillance system in Fiji to collect population-based injury data during a 12-month period. We analysed these data to describe the characteristics of burns resulting in admission to hospital or death in Fiji.

## 2 Methods

### 2.1 Study design and population

The Fiji Injury Surveillance in Hospitals (FISH) system was established in all trauma-admitting hospitals in Viti Levu, the largest island of Fiji. Viti Levu has a population of approximately 600,000 (70% of the resident population of Fiji) and includes both urban and rural areas [7]. Data were collected during a 12-month period (1st October 2005 to 30th September 2006) on all patients admitted to hospital for 12-h or more as a result of injury, or those who died from injury either during or prior to admission. The FISH system was piloted and refinements made prior to the 12 month surveillance period commencing [8]. The pathologists in the two major divisional hospitals conducted post-mortems on all in- and out-of-hospital injury deaths in Fiji during the review period. In the present analysis, cases were limited to those where the main injury was defined as a ‘burn’ and the main mechanism of injury was recorded as due to ‘Fire, heat, electricity’. The ‘heat’ category included injuries as a result of steam, hot liquids, and hot objects.

Researchers and trained nurses located in the hospitals reviewed accident and emergency registers, admission folders, and morgue registers from the surveillance hospitals to identify potential cases. Adapted from the WHO Injury Surveillance Guidelines form [9], a 23-item survey form collected data on: demographic details (name, age, gender, ethnicity); hospital of admission; unique hospital identification number; time and date of injury; admission, and discharge dates; injury event details (place, activity, mechanism, intent, nature of injury, use of alcohol, kava and other substances); details of the injuries sustained and outcomes. The classification of injuries was consistent the International Classification of Diseases and the related schema for coding external causes of injury (ICECI) [10]. A study-specific data dictionary and coding manual provided a standardised protocol for data collection. Activity at the time of injury was classified into three categories: ‘in a conflict situation’, leisure, or work. Injuries that were classified as occurring ‘in a conflict’ situation included dispute(s) between the victim and another person(s). Work-related activities referred to burns sustained during the course of paid employment and excluded injuries that occurred during travel to and from work. All aspects of the surveillance system was piloted, evaluated and refined to assure the quality of data.

Ethnicity was obtained from the medical records of admitted patients or from the mortuary records of cases that died before arrival to hospital. In Fiji, when people register for a National Health Number they select the ethnic group they identify with.

### 2.2 Ethics

Ethical approval for the TRIP project was obtained from the Fiji National Research Ethics Review Committee of the Ministry of Health.

### 2.3 Data analysis

Injury surveillance forms were checked for completeness and verified against the data available in the Patient Information System by the TRIP team. Analyses were conducted using Epi Info Version 3 [11] to provide standard descriptive statistics. The 2007 census information was used to calculate population-based incidence data. Ethnic specific rates were calculated for the two main ethnic groups, Fijian and Indian.

## 3 Results

During the 12-month study period, 116 people were admitted to hospital or died as a result of burns in Viti Levu, accounting for an overall annual incidence of fatal and hospitalised burns of 17.8 per 100,000 population. The characteristics associated with these injuries are presented in Table 1.

### 3.1 Deaths

The 22 fatalities identified corresponded to an annual burn-related mortality rate of 3.4 per 100,000 population. The median age of decedents was 30 years (inter-quartile range 20–55 years). Females accounted for 77.2% of deaths (17/22) and the mortality rate among Indians was 4.3 times that of Fijians (6.1/100,000 vs. 1.4/100,000).

All but one fatality occurred at home, and a third (*n* = 7) of cases died prior to arrival at hospital. Intentional injuries deemed to have occurred following a ‘conflict situation’ accounted for 59.1% (13/22) of deaths, the remainder occurring during leisure activities. All 13 intentional burn fatalities died in hospital, were adult, and of Indian ethnicity. More than three quarters of these cases were female.

### 3.2 Hospitalisations

The 109 people admitted to hospital accounted for an overall admission rate of 16.7 per 100,000 population, and a case fatality rate of 14.0% (15/109). In contrast to fatal burns, the majority (85.3%) of burn-related admissions were unintentional. The median age of admitted patients was 9 years (inter-quartile range 2–37 years) with hospitalisation rates highest among children less than 15 years. Males and females were equally represented among admissions overall with Fijians accounting for a greater proportion of admissions. However, there were important differences in the characteristics of burns by ethnicity, age gender, and mechanism of injury.

The median age of burn admissions was 5 years among Fijians (inter-quartile range 2–21 years) and 29 years among Indians (inter-quartile range 7–44 years). All child burn-related injuries that resulted in admission to hospital were classified as unintentional, and the incidence of hospitalised burns among Fijian children (<15 years) was 1.5 times that of Indians; Indians recorded higher incidence rates in all other age groups. Among admissions of those aged 15 years and older, 68% (34/50) were deemed unintentional and 30% (15/50) were intentional. Of the 15 intentional burn-related admissions, all were adults (13 were Indian and 2 Fijian). The majority of the intentional burn cases died (13/15). The majority of Fijians aged 15 years and older admitted to hospital (17/20) sustained their injuries unintentionally compared to around only half of the Indian cases (16/29). Most burns occurred at home (91.7%), the majority of which occurred during ‘play or leisure activities’ among Fijians and Indians. However, nearly a third of burns among Indians occurred during ‘conflict situations’ – which accounted for all of the self-inflicted burns in this study.

Although a formal burn classification system was not used, the majority of burns were deemed moderate to severe in nature (62.4% and 28.4%, respectively), with less than 10% classified as minor (9.2%). The length of stay for hospitalised cases ranged from 0 to 84 days (median 6 days; inter-quartile range 3–13 days). 29 cases were transferred from smaller sub-divisional hospitals to the two main hospitals in Viti Levu with Burns Units located in Suva and Lautoka. Records for the same patient and burn event were linked to avoid double-counting of incident cases. Four patients were admitted to the Intensive Care Unit, two of whom died.

## 4 Discussion

This population-based study of burns in Fiji resulting in hospital admission or death demonstrates that, taken as a whole, most of these injuries are unintentional, involve children, and occur at home. However, there were important differences in the epidemiology of burns when the data was disaggregated by ethnicity. While Fijian children accounted for most burns in the less than 15 year age group, in all other age groups, Indian women were more likely to be hospitalised or die from self-inflicted burns in circumstances categorised as ‘conflict situations’.

The study employed a prospective population-based approach to data collection, utilising a validated standardised injury surveillance form adapted from the WHO Injury Surveillance Guidelines. However, the study findings need to be interpreted in light of several limitations. The study coding system did not make it possible to determine the source or category of burns (e.g., thermal, chemical, electrical) or identify particular circumstances (e.g., urban–rural location, socio-economic status) that influenced the risks involved. While the injury classification system was broadly aligned to International Classification of Diseases [12], this database did not collect standardised information on the severity of burns. It is also likely that many patients with injuries that did not pose an immediate threat to life were seen in primary care settings or by traditional healers, while others may have been unable to seek medical treatment. It is well recognised that some burns that may not cause an immediate threat to life may nevertheless pose significant risks of disability and disfigurement – which can impact on many aspects of quality of life [13].

While the majority (85%) of burns in the present study were due to unintentional injuries, 59% of fatal burns were deemed to be intentional, almost three quarters of which were among Indian females. A literature review on suicides in Fiji since 1966 published by Morris and Maniam in 2000 [14] identified higher rates among the Indian community relative to the indigenous Fijian population. However, hanging and paraquat poisoning were reported as the main mechanisms with no comment regarding the role of burns. The relatively high rates of suicide among Indian women is consistent with similar findings for this ethnic group in India and several other countries [1,15,16]. Self-immolation is estimated to be responsible for 16–21% of burn-related deaths among women in India [16,17]. A study of burns-related hospital admissions from Sri Lanka found that self-inflicted burns were common among women aged 15–50 years [18]. As in the present study, conflict at home was a common theme in the Sri Lankan study, with the women involved commonly reported to have quarrelled with their husbands or other family members, poured gasoline over their bodies and self-immolated [18]. A 1990 study from Papua New Guinea of 10 women hospitalised following self-inflicted burns by kerosene fire (six of whom died of their injuries) found that all had been in an argument with a person known to the victim [5].

Children accounted for most of the burn-related admissions to hospital, of whom the majority were male. This pattern is consistent with the international literature from both high and LMICs [3,6,19–25]. There are several inter-related issues with regard to the gender and context (e.g., home) of injuries. The predominance of females in all age groups over 15 years reflects a pattern observed in many countries, including Egypt [22], Ethiopia [20], and India [17]. In addition to the previously noted issues relating to self-harm, the higher rates of burns among women in many Asian and African countries are also attributed to local cultural practices such as the use of open fires for cooking, and domestic kerosene appliances such as stoves and lights [2,3,17,20,26].

Burns most commonly occurred at home, which is consistent with other epidemiological studies among both children [3,6,13,21,22,26–28], and adults [19,20,25,26,29,30]. Studies from both HICs and LMICs identify the kitchen as an area of high risk within the home [3,6,19–21,24,26–28,31]. Reports from HIC countries indicate domestic burns are common but at much lower rates than in LMICs [32].

The annual mortality rate in this study was 3.4 per 100,000, similar to rates in other LMICs which are several fold higher than rates in high income countries (approximately 1.0 deaths per 100,000) [32]. While the numbers of fatalities in the present study were insufficient to estimate age-specific rates, the available data are consistent with published research indicating higher fatality rates among older adults [26,33]. In addition to accounting for one of the largest differentials for any injury mechanism, burns constitute the only major injury category with higher mortality rates among women than men [32]. The overall burn mortality rate among Indian females (9.4 per 100,000) in the current study is comparable with that of women living in India (11.3 per 100, 000 per year) who have one of the highest burn mortality rates in the world [34].

Effective interventions for burns prevention and management in LMICs constitute a focus of increasing global attention given the disproportionate burden and significant unmet needs in these settings [1], particularly given the lack of resources and technology to comprehensively treat, rehabilitate, and provide adequate community-based services for individuals surviving serious burns [35]. The World Health Organisation has worked collaboratively with burn experts worldwide to develop an evidence-based global strategy for burn prevention and care [1].

The findings from the present study indicate that Pacific Island nations would benefit from the development of comprehensive burns prevention and control strategies that address the major contributing factors and optimise treatment, rehabilitation and support programs that enhance recovery. Future research should particularly focus on better understanding the characteristics that constitute proximal factors (e.g., cooking practices, partner abuse), as well as the wider socio-economic and ethno-cultural determinants that increase both the exposure to such injuries. The present study particularly highlights the potential of aggregate data at the country or regional level to mask important differences in sub-groups within countries. The contextual issues involved require particular attention with more targeted community-based strategies.

## Conflict of interest statement

No conflict of interest exists between any of the authors and this research.

## Figures and Tables

**Table 1:** Characteristics of burns resulting in death or hospital admission in Viti Levu, Fiji, October 2005–September 2006.

Characteristics	Total deaths (*n* = 22)	Hospitalisations^b^ (*n* = 109)	Total cases (*n* = 116)
	*n* (%)	*n* (%)	*n* (%)
Gender			
Male	5 (22.7)	57 (52.3)	59 (50.9)
Female	17 (77.2)	52 (47.7)	57 (49.1)
Age group (years)			
0–14 years	a	59 (54.1)	61 (52.6)
15–29 years	8 (36.3)	16 (14.7)	18 (15.5)
30–44 years	a	17 (15.6)	17 (14.7)
45 years or more	8 (36.3)	17 (15.6)	20 (17.2)
Ethnicity			
Fijian	5 (22.7)	63 (57.8)	47 (40.5)
Indian	16 (72.7)	44 (40.4)	66 (56.9)
Other	1 (4.5)	a	a
Activity at time of injury			
In a conflict situation	13 (59.1)	16 (14.7)	17 (14.7)
Leisure	8 (36.4)	78 (71.5)	83 (71.6)
Work	a	9 (8.3)	10 (8.6)
Other/unknown	0	6 (5.5)	6 (5.2)
Place where injury occurred			
Home	21 (95.5)	100 (91.7)	107 (92.2)
Work	0	5 (4.6)	5 (4.3)
Other/Unknown	a	4 (3.7)	4 (3.4)
Intent			
Unintentional	9 (40.9)	93 (85.3)	99 (85.3)
Intentional	13 (59.0)	15 (13.8)	16 (13.8)
Unknown	0	a	a
Severity of Injury			
Minor	0	10 (9.2)	10 (8.6)
Moderate	0	68 (62.4)	68 (58.6)
Severe	22 (100)	31 (28.4)	38 (32.8)
Outcome			
Discharged	0	94 (86.2)	94 (81.0)
Died	22 (100)	15 (13.8)	22 (19.0)

a Less than four subjects per cell; data omitted.

b 15 cases died in hospital.
